# Standardized *in-vitro* evaluation of CAR-T cells using acellular artificial target particles

**DOI:** 10.3389/fimmu.2022.994532

**Published:** 2022-10-20

**Authors:** Rona Harari-Steinfeld, V. S. S. Abhinav Ayyadevara, Lizette Cuevas, Francesco Marincola, Kyung-Ho Roh

**Affiliations:** ^1^ Refuge Biotechnologies, Menlo Park, CA, United States; ^2^ Biotechnology Science and Engineering, The University of Alabama in Huntsville, Huntsville, AL, United States; ^3^ Department of Chemical and Materials Engineering, The University of Alabama in Huntsville, Huntsville, AL, United States

**Keywords:** CAR-T, *in-vitro* assay, potency assay, acellular, biomaterials, artificial, target cell

## Abstract

The horizon of immunotherapy using CAR-T cells is continuously extending to treat solid tumors beyond the success in the treatment of liquid tumors. Precise *in-vitro* evaluations of CAR-T cells for their phenotypes, quantity and quality of activation in various tumor microenvironments including different antigen densities, and the resulting effector functions are critical for the successful development of CAR-T therapies and safe translation to clinics. Unfortunately, the development of methods and tools to accommodate these needs have been lagging behind. Here, we developed a novel biomaterial platform, acellular artificial target particles (aaTPs) against CAR-T cells, using magnetic microbeads that are already widely employed in the manufacturing of T cell products. By devising a simple and standardized procedure, we precisely controlled the antigen surface densities presented on the aaTPs for a wide range. By co-incubation of aaTPs with CAR-T cells followed by flow cytometry and cytokine assays, we quantitatively determined the antigen-specific and dose-dependent activation of anti-HER2 CAR-T cells. We also demonstrated that the aaTP can serve as a clean target cell in *in-vitro* assays to prove the proposed mechanism of action of a next-generation CAR-T product. Overall, the simple, inexpensive, modular and precisely controllable synthetic nature of aaTPs enables the development of clean and standardized *in-vitro* assays for CAR-T cells, which provides critical advantages over the conventional assays using target cell lines. The design of aaTPs can be extended to include other tumor antigens and relevant surface molecules of physiological target cells. Thus, the aaTP platform has great potential as a standardized tool for the development and evaluation of both conventional and new CAR-T products in the context of approval from regulatory agencies and clinical translation.

## Introduction

Adoptive cell transfer therapies using T cells modified with chimeric antigen receptors (CARs) have had tremendous success in the treatment of multiple B cell malignancies (1). In the treatment of solid tumors, however, success at an equivalent level has not been observed yet due to unique challenges such as lack of truly specific antigens and highly immunosuppressive tumor microenvironments. In order to overcome such challenges and enhance the safety and efficacy of CAR-T therapies on solid tumors, various strategies have been developed to produce next-generation CAR-T therapeutics (2).

For any CAR platform, a careful examination on the activity, specificity, and potency of engineered cells is required as product characterization and release testing prior to use in the clinic. Currently, a majority of the *in-vitro* assays mainly relies on the co-culture of CAR-T cells with model tumor cell lines that express the target-antigens accompanied by flow cytometry-based and/or cytokine analyses (e.g. Luminex^®^ or ELISA). However, there are some significant limitations in the current assay designs employing model target cell lines. First, the antigen densities expressed by the target cell lines are often heterogeneous, unstable, difficult to control, thus very challenging to standardize. As the activation of CAR-T cells strongly depends on the expression level of target antigens on tumor cells (3–5), *in-vitro* assays using non-standardized antigen-densities pose intrinsic limitations. Second, as the dynamics of the interaction between target cell line and CAR-T cells cannot fully mimic the physiological tumor cells with its own physiology, the outcomes of *in-vitro* assays are often difficult to directly compare to *in-vivo* or in-patient efficacy. Third, as the target cells change in number over the course of an assay (*i.e.* proliferating or killed), a precise evaluation of CAR-T functions (e.g. cytokine secretion) based on the antigen-dosage (*e.g.* effector to target (E:T) ratio) is difficult to achieve. Fourth, as each target cell line has its own genetic and epigenetic background and the corresponding phenotypes, their interactions with the CAR-T cells would be inevitably affected by a certain set of such cell-line-specific unknown factors, which may become a hurdle for assay standardization. Lastly, the co-presence of target cells and CAR-T cells in an *in-vitro* assay make it intrinsically challenging to extract CAR-T-cell-specific information from high-throughput genetic and epigenetic sequencing experiments.

As an alternative to the model target cell lines, here we report a biomaterial-based acellular artificial target particles (aaTPs) for standardized *in-vitro* potency evaluation of CAR-T cells. We employed an iron-oxide microparticle (Dynabeads™) as the biomaterial platform because the same microbeads coated with anti-CD3 and anti-CD28 antibodies are already widely employed in clinical manufacturing (activation and expansion) of CAR-T cells and other therapeutic T cells. We developed the aaTPs to present the human epidermal growth factor receptor 2 (HER2) in a precisely controlled manner in order to employ these in standardized potency assays for CAR-T cells against HER2. HER2 is a well-established therapeutic target protein due to its overexpression in various solid tumors such as the breast, colon, gastric, ovary, head and neck, lung, bladder, uterine cervix, and esophageal cancers (6). A humanized murine monoclonal antibody, 4D5 (trastuzumab, Herceptin^®^), has been clinically used for treatment of HER2+ breast cancer. More recently, CAR-T cells against HER2+ cancers have been developed and investigated at preclinical and clinical stages (7–9).

Here, we first demonstrate how we developed a series of aaTPs that present precisely controlled densities of HER2 over a wide range. And we will use these in various *in-vitro* assays that are designed to examine the antigen-dependent CAR-T cell activation and secretion of cytokines for conventional CAR-T cells expressing anti-HER2 CAR (4D5). Lastly, we also demonstrate how to utilize the standardized assays for *in-vitro* evaluation of novel functions of one of the next-generation CAR-T products. The aaTPs are cheap, require minimal maintenance, and serve as effective reagents that can provide a clean, controlled, and antigen-dependent activation of CAR-T cells for their *in-vitro* potency assays and manufacturing process.

## Materials and methods

### Protein quantitation by BCA assay

Concentrations of all protein solutions were measured using the BCA protein assay kit (Pierce) in NanoDrop One^C^ (Thermo Scientific). All samples, including the BSA standards, were incubated at 60°C for 30 minutes in a water bath before taking measurements.

### Modifications of HER2 and biotin quantification

For labeling of HER2 with fluorescent dye molecules, recombinant human HER2 ectodomain (70.2 kDa, Acro Biosystems) was originally reconstituted with ultrapure water and further diluted in 80 mM sodium bicarbonate buffer (pH 8.3). For 50 μg of HER2 proteins, 21.4 nmol of freshly prepared fluorescent dye, Alexa Fluor 647 (AF647), with succinimidyl ester (Invitrogen) was added. The reactions were allowed to occur overnight at 4°C on a microtube rotator. The reaction mixture was purified by exchanging the buffer into PBS containing 0.02% (w/v) NaN_3_ (pH 7.4) using Zeba Spin Desalting Columns (7 kDa MWCO, Thermo Scientific). The labeled HER2 solutions were stored at 4°C until used. For biotinylation of HER2, the same recombinant human HER2 ectodomain was reacted with a 20-molar excess of NHS-PEG_4_-Biotin (Thermo Scientific, NPB) in 0.1 M sodium bicarbonate (Electron Microscopy Sciences) buffer at pH 8.3 in 4°C for 2 hours on a tube rotator. After the reaction, the biotinylated protein was purified by exchanging the buffer into PBS using Zeba Spin Desalting Columns (7 kDa MWCO, Thermo Scientific). Post-purification, the number of biotins per protein molecule was immediately confirmed by HABA assay (Biotin Quantitation Kit, Pierce) by following the manufacturer’s recommended protocol. When the molar ratio of biotin to HER2 is confirmed to be within the range between 7 and 8, we used the biotinylated HER2 for preparation of acellular artificial target particles.

### Loading of HER2 onto dynabeads for preparation of acellular artificial target particles

The main stock of streptavidin-coated superparamagnetic microbeads (Dynabeads^®^ M280 Streptavidin, Thermo Fisher, hereafter just “M280”) were vigorously vortexed for 30 seconds just before use. Typically, 100 μl of M280 beads (10 mg/ml stock) were taken into a 1.5 ml microcentrifuge tube and resuspended in 1 ml of Phosphate Buffered Saline (PBS, pH 7.4). The M280 beads were washed twice with magnetic precipitation by placing the suspension on a magnetic separator (Invitrogen DynaMag™-2 Magnet) for 1 minute and discarding the supernatant for every washing step. Into the M280 beads resuspended in 1 ml of PBS, varying amount of biotinylated HER2 (0.1 – 10 μg) or biotinylated BSA (20 μg) were added and immediately vortexed. The tubes were covered with parafilm and kept overnight at 4°C on a tube rotator. Upon completion of incubation, the beads in each microtube were briefly centrifuged, resuspended, and washed once after magnetic precipitation. Then, onto the resuspended HER2 beads in 1 ml of PBS, 20 μg of biotinylated BSA was added and immediately vortexed. Again, each tube was covered with parafilm and kept at 4°C on a tube rotator for 1 hour. Upon completion of incubation, the beads in each microtube were briefly centrifuged, resuspended, and washed three times with PBS *via* magnetic precipitation steps. The final M280 microbeads were resuspended at a concentration of 10 mg/ml in PBS containing 0.02% (w/v) sodium azide and stored in 4°C.

### Evaluation of HER2 surface densities by standardized flow cytometry

The surface densities of HER2 molecules on the artificial target particles were examined by standardized flow cytometry using a commercial quantitative analysis kit, QIFIKIT^®^ (Agilent) following the user manual provided by the manufacturer of the kit. Briefly, we first set up and optimized the voltages for forward-scattering (FSC), side-scattering (SSC), and BL1 fluorescence channel (for FITC) in Attune NxT Flow Cytometer (Invitrogen) using the set-up beads (QIFIKIT^®^). Next, following standard staining and washing procedures, we stained the calibration beads (QIFIKIT^®^), a combination of five populations of beads bearing different known numbers of mouse antibodies on their surfaces, with FITC-conjugated goat anti-mouse immunoglobulin F(ab’)_2_ fragment. Then, using flow cytometry, the mean fluorescence intensities (MFIs) of the calibration beads of known surface densities, represented by antigen binding capacity (ABC) values, were recorded, from which a standard curve between the MFI and ABC values was constructed. For the measurement of HER2 densities of artificial target particles, typically 10^5^ microbeads were stained with 500 ng of mouse anti-human HER2 antibody (clone 191924) in 50 μl of BSA buffer (PBS supplemented with 0.1% BSA) for 1 hour at 4°C on a tube rotator. After washing with FACS buffer (PBS, 0.5% BSA, 2 mM EDTA) once by centrifugation, the microbeads were stained with 500 ng of FITC-conjugated goat anti-mouse immunoglobulin F(ab’)_2_ fragment in 50 μl of FACS buffer for 45 minutes at 4°C on a tube rotator. Finally, the microbeads were washed twice with FACS buffer by centrifugation. After resuspending the microbeads in 300 μl of FACS buffer, the ABC values were measured at the same voltage settings used for obtaining the corresponding calibration curve in the same flow cytometer.

### Primary T cell isolation and CAR T cell production

Healthy donor leukopak were purchased from PAA Research Group (Johnson City, TN). Fresh peripheral blood mononuclear cells (PBMCs) were isolated by low-density centrifugation on Lymphoprep (Stem Cell Technology) according to the manufacturer’s instructions. Pan T cells were isolated using Dynabeads Untouched Human T Cells (Invitrogen). To generate CAR-T cells, at day 0 cryopreserved Pan T cells were thawed and activated in 24-well plate coated with human anti-CD3 (OKT3, 1 µg/ml, Biolegend) and human anti-CD28 (CD28.2, 1 µg/ml, BD Pharmingen™) antibodies in T cell culture medium [RPMI supplemented with 10% human serum, 2 mM GlutaMAX, 50 µM 2-Mercaptoethanol (Gibco), 100 U/ml penicillin and 100 µg/ml streptomycin (Gibco)]. Both recombinant human IL-7 and IL-15 (Gibco) were provided at 10 ng/ml. For conventional CAR-T engineering, T cells were transduced with lentiviral vectors carrying HER2-CAR-tNGFR on day 2 after activation. For next generation CAR-T engineering, T cells were transduced with lentiviral vectors carrying LAT-dCas9KRAB-Q8 at day 1 and HER2 CAR-TEV-tNGFR/PD-1sg at day 2 after activation (**Supplementary Figure 1**). At day 5, the T cells were transferred into a tissue-culture-treated 24-well plate. At day 6, transduced T cells were enriched through cell sorting (Sony Cell Sorter) tNGFR positive cells for conventional CAR and Q8 and tNGFR double positive for next generation T cells. After cell sorting, T cells were maintained at 0.5 − 2 × 10^6^ cells per ml in T cell culture medium with IL-7 and IL-15 (10 ng/ml). Cells were harvested for cryopreservation at day 14-21 of the manufacturing run.

### FaDu cell culture

The human head and neck squamous cell carcinoma line FaDu was obtained from ATCC (Manassas, VA). FaDu cells were maintained in DMEM complete media (DMEM supplemented with 10% FBS, 100 U/ml penicillin and 100 μg/ml streptomycin, Gibco). When the plates are confluent, the cells were detached by trypsin-EDTA, and split at 1:5 ratio for sub-cultivation. All cells were routinely tested for potential mycoplasma contamination using the MycoAlert Mycoplasma Detection Kit (Lonza).

### Bead (aaTP)-cell co-incubation assays

Engineered T cells were thawed and rested overnight in the T cell culture medium with human IL-7 and IL-15 (10 ng/ml). Then, 50,000 CAR-T cells were plated in 96-well round bottom plates in T cell media with no added cytokines, and HER2 beads (aaTPs) were added at the described bead:cell ratios (5:1-1:125, 250,000-400 beads), to each well. Incubations were maintained for 24-72 before further analyses.

### Flow cytometry analysis

24-72 hours after co-incubation with beads, CAR-T cells were collected and stained with the following antibodies NGFR-FITC (clone ME20.4, Biolegend), Q8 (clone QBEND/10, ThermoFisher), CD45-AF700 (clone HI30, Biolegend), CD3-APC (clone SK7, Biolegend), CD4-PerCP (clone SK3, Biolegend), CD8-BV510 (clone SK1, Biolegend), PD-1-BV421 (clone EH12.2H7, Biolegend), CD69-BV605 (clone FN50, Biolegend), and HER2-PE-cy7 (clone 24D2, Biolegend). Live/dead discrimination was achieved using LIVE/DEAD fixable Near-IR dead cell stain kit (ThermoFisher). Flow cytometry results were analyzed using Kaluza software (Beckman Coulter).

### Cytokine measurement (ELISA)

24-72 hours after co-incubation of beads (aaTPs) and CAR-T cells, culture supernatants were collected and analyzed for IFN-γ, IL-2 and TNF-α by ELISA according to the manufacturer protocol (Biolegend).

### Graphical representation and statistical analysis

All graphs were plotted and all the statistical analyses were performed using Prism software (v9.0, GraphPad). Flow cytometry data were analyzed and plotted using FlowJo software (v10).

## Results

### Generation of acellular artificial target particles (aaTPs) that present HER2 molecules at controlled varying densities

As a model CAR, we first employed the second-generation CAR that contains an anti‐human epidermal growth factor receptor 2 (HER2) single chain variable fragment (scFv) with CD28 and CD3ζ signaling domains. The scFv is from monoclonal anti-HER2 antibody clone 4D5, the humanized version of which has been used for treatment of HER2+ breast cancers (10–12). To generate acellular artificial target particles (aaTPs) for this model CAR-T against HER2, we selected the streptavidin-coated superparamagnetic iron-oxide microbeads of 2.8 μm in diameter (Dynabeads, hereafter “M280”) as our platform. This microbead platform has been widely and successfully employed in manufacturing of various cell therapeutics.

In order to conjugate the HER2 molecules onto the streptavidin-coated surface of M280 beads, we developed a suitable strategy for biotinylation of the recombinant HER2. Among many bioorthogonal chemistries, we tested the feasibility of employing the specific reaction between the succinymidyl ester and primary amine group of lysine residues. In fact, the extracellular domain of human HER2 protein contains 13 lysines, and only two lysine residues (K569 and K593) are in the vicinity to 4D5 binding epitope, based on the crystal structure of human HER2 complexed with 4D5 Fab (**Supplementary Figure 2**, PDB ID: 1N8Z). It is likely that the 4D5 epitope will be well preserved and effectively presented to 4D5 CARs on CAR-T cells when one of the 11 (out of 13 total) lysine residues are used for conjugation. In order to test this hypothesis, we first conjugated the recombinant HER2 with fluorescent dye Alexa Fluor 647 (AF647) using the same chemistry. In flow cytometry the recombinant HER2 ectodomain labeled with fluorescent dyes (HER2-AF647) successfully stained the 4D5 CAR-expressing cells specifically and in a dose-dependent manner (**Supplementary Figure 3**). This indicates that the conjugation strategy targeting random lysine residues of HER2 can be employed without abrogating recognition of HER2 by 4D5 CAR. So, we biotinylated HER2 using NHS-PEG4-Biotin (NPB), which provided additional flexibility onto the conjugation link with 4 repeating units of ethyleneglycol (PEG4). Typical biotinylation reaction of HER2 with 20-molar excess of NPB yielded biotin-to-HER2 ratio of 7-8, which was confirmed by HABA assay (Materials and Methods).

**Figure 1:**
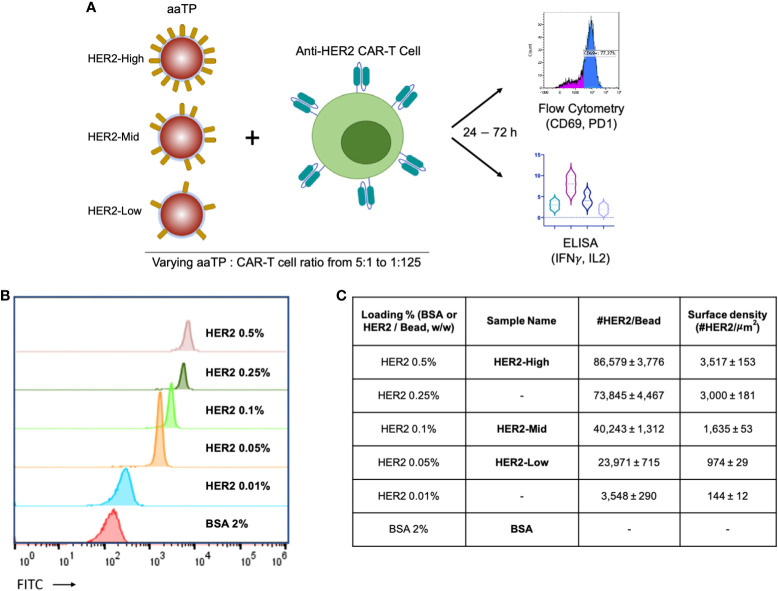
Design of the acellular artificial target particles (aaTPs). **(A)** Schematic diagram of *in-vitro* assays of anti-HER2 CAR-T cells using aaTPs presenting HER2 at varying surface densities; **(B)** Flow cytometry histograms of aaTPs prepared by incubation with varying amount of HER2; **(C)** A table of aaTPs summarizing the corresponding loading condition, sample name, number of HER2 molecules per aaTP (bead), and surface density. Data values in **(C)** represent mean ± standard deviation of 3 independent measurements.

It has been previously demonstrated that the level of CAR-T cell activation and resulting efficacies and toxicity are critically dependent on the antigen densities on target cells (3–5). Thus, it is highly desired to design a series of aaTPs that present antigens at a wide range of controlled densities (**Figure 1A**). Here, we tried to achieve this goal by systematically varying the relative mass percentage of biotinylated HER2 compared to M280 microbeads during the incubation (loading) period. When these HER2-loaded beads were analyzed by flow cytometry after being serially stained with a primary antibody against human HER2 and a FITC-conjugated secondary antibody, their mean fluorescent intensity (MFI) values varied in correlation with the incubation mass percentage (**Figure 1B**). By comparing these MFI values to those of standard beads of known antigen binding capacity (ABC) values (QIFIKIT®, see Materials and Methods), we could determine the physiologically relevant HER2 surface density on each aaTP population (**Figures 1B, C**). By varying the incubation mass percentage from 0.01% to 0.5% (50 fold), we could vary the number of HER2 molecules per microbead from 3,548 to 86,579 (24 fold). The loading of HER2 molecules onto the microbeads showed a typical saturation behavior (**Supplementary Figure 4**), showing that the measured HER2 surface density reaches its maximum value around 0.5% (w/w). It is worth mentioning that the HER2 surface densities reported here (ABC values) do not necessarily represent the entirety of HER2 molecules conjugated to the surface but rather represent the number of HER2 molecules that can be recognized by the antibody molecules employed in the assay. Nevertheless, we successfully created the aaTPs that present HER2 molecules at widely varying physiologically relevant densities. We selected three, the microbeads from 0.05, 0.1 and 0.5 incubation mass percentage, and named them HER2-Low, -Mid, and -High, respectively, for the following investigations (**Figure 1C**).

### aaTPs largely maintain T cell viability and mimic patterns of activation induced cell death

**Figure 2:**
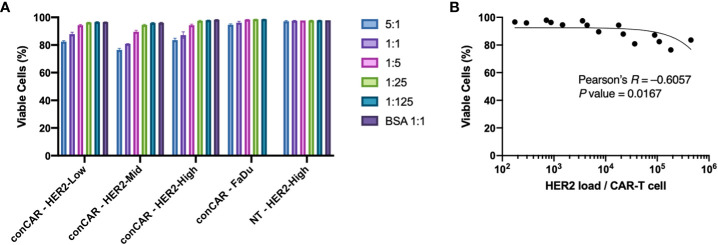
Biocompatibility of the acellular artificial target particles (aaTPs). **(A)** Viability of anti-HER2 CAR-T cells (conCAR) and non-transduced T cells (NT) incubated with aaTPs of varying HER2 surface densities at varying target (aaTP)-to-effector (CAR-T cell) ratio for 24 hours. Each bar graph with an error bar represents mean and standard deviation of n = 3 replicates. **(B)** Correlation between the viability of anti-HER2 CAR-T cells and antigenic dosage with the Pearson’s *R* and the *P* value (two-tailed).

We first investigated the biocompatibility of the aaTPs. We incubated HER2-Low, -Mid, -High, the negative control (BSA coated M280) beads, and FaDu cells with either conventional anti-HER2 CAR-T cells or non-transduced (NT) T cells at varying beads (aaTPs) to CAR-T cells ratio of 5:1, 1:1, 1:5, 1:25, and 1:125. As a positive target-cell control, we used FaDu, a squamous carcinoma derived cell line expressing HER2 that is commonly used as a model cell line for HER2+ tumors. For direct comparison, we measured the number of HER2 molecules expressed by FaDu cell using QiFi assay and it was 10,508 (ABC value per cell) which corresponds to approximately 15 HER2 per μm^2^ as the average size of FaDu cell is 15 μm in diameter. The BSA-only beads did not affect the viability of either conventional anti-HER2 CAR-T cells or non-transduced (NT) T cells, which indicates that the microbead platform of aaTPs itself is biologically inert to the T cells (**Figure 2A**). For HER2-presenting aaTPs, we observed a mild reduction in CAR-T cell viability as the bead-to-cell ratio increases for all HER2-presenting groups, *i.e.* HER2-Low, -Mid, and -High, while NT cells were not affected (**Figure 2A**). Increasing ratio of FaDu cell to CAR-T cell also brought upon slight decreasing CAR-T cell viability, but the effect was not as pronounced as aaTPs (**Figure 2A**), which is likely due to the lower HER2 density on FaDu cells compared to aaTPs. In fact, multiplying number of HER2 molecules per target cells (or particles) and the ratio of target cell (or particle) to CAR-T cell, the number of HER2 molecules per CAR-T cell can be calculated as antigen-dosage. As shown in **Figure 2B**, the viable CAR-T cell percentage decreased as the overall antigen-dosage increased (Pearson’s *R* = −0.6057, *P* value = 0.0167). Therefore, this antigen-specific and dose-dependent cytotoxicity is most likely a result of the specific interactions between the HER2 and CAR and the subsequent signaling events, which can be explained by activation induced apoptosis of CAR-T cells (13).

### aaTPs efficiently induce antigen-specific activation of CAR-T as indicated by T-cell activation markers

**Figure 3:**
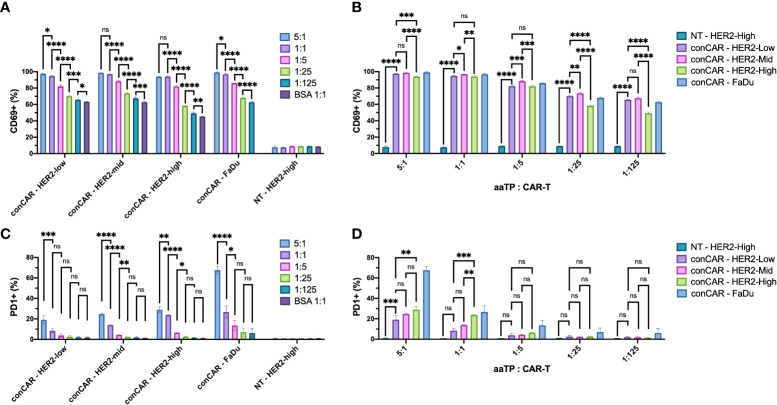
Expression of activation markers, CD69 **(A, B)** and PD1 **(C, D)**, on anti-HER2 CAR-T cells (conCAR) and non-transduced T cells (NT) measured by flow cytometry after co-incubation with aaTPs of varying HER2 surface densities at varying target (aaTP)-to-effector (CAR-T cell) ratio for 24 hours. Each bar graph with an error bar represents mean and standard deviation of n = 3 replicates. Statistical significance from ordinary one-way ANOVA followed by Tukey’s multiple comparisons test were represented as * (*P* < 0.0332), ** (*P* < 0.0021), *** (*P* < 0.0002), **** (*P* < 0.0001), and ns (not significant).

To further test the ability of aaTPs to stimulate the activation of CAR-T cells, we incubated all aaTPs and FaDu cells for 24 hours and measured the expression of canonical early- and mid-activation markers, CD69 (14) and PD1 (15), respectively, *via* flow cytometry. As expected, all HER2+ aaTPs as well as FaDu induced increase in CD69+ population, and this induction was positively correlated with the bead-to-cell ratio (e.g. more HER2 particles produced more CD69+ cells) (**Figure 3A**). For HER2-Mid and HER2-High, the 5:1 and 1:1 bead-to-cell ratios did not show statistically significant differences, which is probably due to the fact that the readout (i.e. CD69+ percentage) is saturated to close to maximum 100%. Cells without CAR expression (NT) did not show similar increase in CD69 expression upon incubation even with HER2-High beads, thus the overexpression of CD69 in CAR-T cells is a result of CAR signaling. In order to compare the data according to the HER2 densities for an identical bead-to-cell ratio, we plotted the same data set differently in **Figure 3B**. Again, as expected, HER2-Mid yielded an equal or higher percentage of CD69+ cells than HER2-Low. However, HER2-High yielded lower percentages of CD69+ cells than HER2-Mid in all bead-to-cell ratios, which was an unexpected trend. It was also interesting to observe, that the BSA-only beads could induce slight increase in CD69 expression in CAR-T cells. In our preliminary experiments, our CAR-T cells repeatedly demonstrated slightly elevated expression of CD69 even without any stimulation and the addition of BSA-beads did not incur any effect (**Supplementary Figure 5**). Thus, this slight increase in CD69 level is likely a manifestation of basal level of activation from CAR-mediated tonic signaling (16, 17), and we kept BSA-only bead as our negative control for further investigations.

Similarly, we examined the expression of PD1 (**Figures 3C, D**). A similar HER2-dose dependent increase in PD1+ population was observed although it was not as robust as we have observed with CD69. A significant increase in PD1+ percentage was observed in target-to-CAR-T-cell ratio of 5:1 compared to 1:1 in all HER2-presenting beads and FaDu group (**Figure 3C**). Meanwhile 1:1 ratio yielded significantly higher PD1+ percentages than 1:5 ratio only in HER2-Mid, HER2-High, and FaDu groups, and 1:5 ratio was significantly higher than 1:25 ratio only in HER2-Mid and HER2-High groups (**Figure 3C**). In comparisons between HER2-presenting beads of different densities, only HER2-High yielded higher PD1+ percentage compared to HER2-Low only in 5:1 and 1:1 bead-to-cell ratios (**Figure 3D**). HER2-High was significantly higher than HER2-Mid only in 1:1 bead-to cell ratio (**Figure 3D**). It is worth noting that PD1+ population increased more pronouncedly by FaDu cells than by aaTPs, even if the HER2 density of FaDu is much lower than aaTPs. This could be attributed to the difference in size and interaction dynamics between FaDu and aaTPs or unknown effects of other molecules on FaDu.

Altogether, the dynamic ranges in signal readout (*i.e.* marker+ percentages) for canonical early- and mid-activation markers, CD69 and PD1, respectively, were very different for the same HER2 dosages. At the time of measurement (24 hours), all CD69+ percentages were higher than 40%, while all PD1+ percentages were below 30% for all tested HER2 dosages presented by aaTPs. For expression of CD69, the best dose-dependent comparisons were made using HER2-Low and lower bead-to-cell ratios, because HER2-Mid and -high reached the saturation in readout near the maximum bead-to-cell ratios. In comparison, statistically meaningful comparisons in PD1+ percentages were better achieved by HER2-Mid and -High than HER2-Low and higher bead-to-cell ratio than 1:5, because lower HER2 dosages did not yield high enough readouts. Nevertheless, the precisely dose-dependent (either by changing bead-to-cell ratio or by varying the HER2 density) CD69 and PD1 expressions in cognate CAR-T cells achieved by a series of aaTPs strongly demonstrate the great potential of this system as a clean toolkit for potency measurement and mechanism of study of CAR-T products.

### aaTPs are able to induce effector functions of CAR-T cells *in vitro* as demonstrated by measuring cytokine secretion

**Figure 4:**
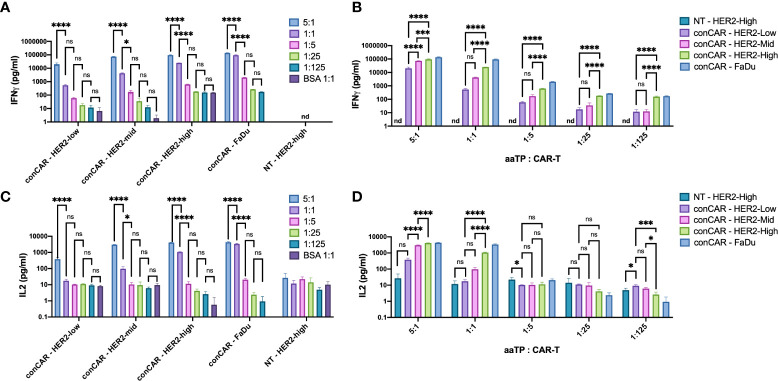
Secretion of cytokines, IFNγ **(A, B)** and IL2 **(C, D)**, by anti-HER2 CAR-T cells (conCAR) and non-transduced T cells (NT) measured by ELISA after co-incubation with aaTPs of varying HER2 surface densities at varying target (aaTP)-to-effector (CAR-T cell) ratio for 24 hours. Each bar graph with an error bar represents mean and standard deviation of n = 3 replicates. Statistical significance from ordinary one-way ANOVA followed by Tukey’s multiple comparisons test were represented as * (*P* < 0.0332), *** (*P* < 0.0002), **** (*P* < 0.0001), and ns (not significant).

As a result of activation, CAR-T cells secrete various cytokines, to support their own functions as well as induce signaling in neighboring cells (such as tumor cells and TME cells). These cytokines, especially IFN(γ) and IL2, are considered the hallmark metrics for measuring T-cell potency in the CAR-T field (18). Here, we tested if such potency assay can be performed using the aaTPs instead of tumor cells. After 24 hours of incubation, all HER2+ aaTPs and FaDu effectively induced significant increases in secretion of both IFNγ and IL2 (multiple orders of magnitude higher than their basal levels) at target-to-CAR-T-cell ratio of 5:1 (**Figures 4A, C**). HER2-Mid, HER2-High, and FaDu effectively induced significant increase in secretion of both IFNγ and IL2 at target-to-CAR-T-cell ratio of 1:1 as well, but the concentrations were significantly lower than those from 5:1 ratio (**Figures 4A, C**). Minimal levels of cytokine secretion were observed when anti-HER2 CAR-T cells were incubated with BSA-only beads, and minimal to non-detectable (nd) levels (IFNγ, under assay lower limit of detection) were observed when non-transduced T cells were incubated with HER2-High, which collectively clearly indicates that the observed cytokine secretions are induced by the activation of CAR-T by the target antigen, HER2. In terms of sensitivity on different densities, IFNγ assay was much more sensitive than IL2 assay (**Figures 4B, D**). In fact, HER2-High yielded significantly higher IFNγ concentrations than HER2-Mid on all tested bead-to-cell ratios, and even HER2-Mid yielded significantly higher IFNγ concentration than HER2-Low on 5:1 ratio (**Figure 4B**). In comparison, HER2-High yielded significantly higher IL2 concentrations than HER2-Mid only on bead-to-cell ratios of 5:1 and 1:1, and HER2-Mid yielded significantly higher IL2 concentration than HER2-Low only on 5:1 ratio (**Figure 4D**).

### The expression of activation markers CD69 and PD1 and the secretion of cytokines IL2 and IFNγ were antigen dose-dependent with their own minimal dosage thresholds

**Figure 5:**
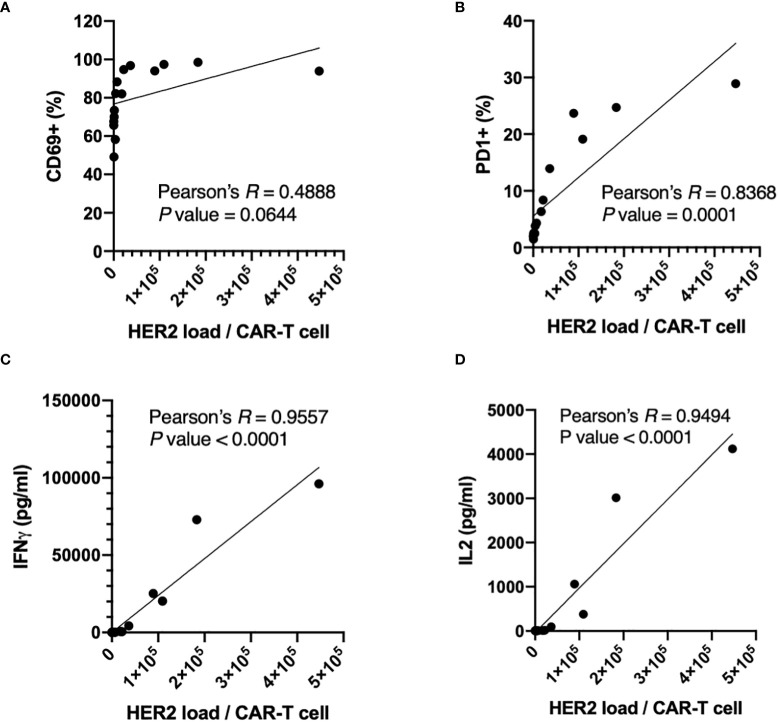
Correlations between expression of activation markers, CD69 **(A)** and PD1 **(B)**, or secretion of cytokines, IFNγ **(C)** and IL2 **(D)**, of anti-HER2 CAR-T cells and HER2 dosages per CAR-T cells with the Pearson’s *R* and the *P* values (two-tailed).

The activation (**Figure 3**) and potency (**Figure 4**) assays showed clear trends in general: the positive population of activation markers and the concentrations of secreted cytokines increased as HER2 surface densities increased from HER2-Low to -Mid to -High, or as target particle to CAR-T cell ratio increased. In fact, the HER2 antigen dosage per CAR-T cells can be calculated by multiplying HER2 surface densities on targets and target to CAR-T cell ratio. Thus, we carefully examined the correlation between the activation or potency parameters and HER2 antigen dosage per CAR-T cells (**Figure 5**). As we expected, all measured parameters, *i.e.* the size of positive populations of activation makers (CD69 and PD1) and the concentration of secreted cytokines (IFNγ and IL2) showed positive correlations with antigenic dosage, as indicated by Pearson’s R values (**Figure 5**). However, the shapes of correlation were quite different. CD69+ percentages reached its maximum values at relatively low antigen dosage (approximately 3 x 10^4^), which made the correlation above this antigen dosage quite flat. Thus, the *P* value (0.0644) of correlation calculated for the entire range of antigen dosage yielded a non-significant level (**Figure 5A**). In comparison, the positive percentages of a later activation marker PD1 showed stronger correlation for the entire range of antigen dosage (Pearson’s *R* = 0.8368, *P* value = 0.0001), and the biggest dynamic range of PD1+ percentage was observed at HER2 dosage between 2 x 10^4^ and 2 x 10^5^ (**Figure 5B**). Furthermore, the correlations of potency parameters (secretion of IFNγ and IL2) were even more strong with much higher significance (**Figures 5C, D**). These parameters showed dynamic dose-dependency for the entire range of the antigen dosage above around 2 x 10^4^ HER2 molecules per CAR-T cells. It is important to notice that different parameters show different dynamic ranges for different antigen-dosage thresholds. Therefore, the antigen-specific activation-induced *in-vitro* potency assay should be designed in a parameter-specific manner to maximize the dynamic range (i.e. signal-to-noise ratio) with a properly controlled antigenic dosage. Such a highly customized assay will be enabled by precise control over antigenic dosage by standardized aaTPs. Unlike the conventional target cell line, these assays can be performed without concerns of varying antigen dosages over incubation period due to killing or proliferation of target cells.

### The use of aaTPs extends to standardized assay for novel mechanism of action of next-generation CAR-T cells

**Figure 6:**
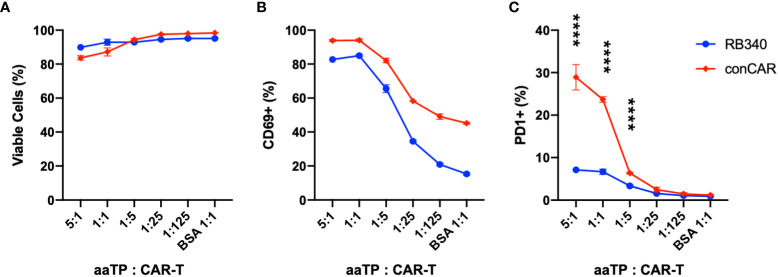
Comparison of conventional CAR-T (conCAR) and a next-generation CAR-T (RB340). Viability **(A)** and upregulation of CD69 **(B)** or PD-1 **(C)** were measured after co-incubation of these anti-HER2 CAR-T platforms with HER2-Mid aaTPs at varying target (aaTP)-to-effector (CAR-T cell) ratio for 24 hours. Each data point with an error bar represents mean and standard deviation of n = 3 replicates. In **(C)**, statistical significance from student’s t-test were represented as **** (*P* < 0.0001).

We next tried to apply aaTPs in the characterization of a more complex CAR-T cell product. RB-340 is a ‘next-generation’ CAR-T cell in which activation of the CAR receptor by its respective target antigen (HER2) not only stimulates T-cell activation but also inhibits upregulation of PD-1 using CAR-dependent activation of dCAS9-KRAB element which specifically targets PD-1 (19). Upon co-incubation with HER2-Mid aaTPs, both RB-340 and conventional CAR-T cells showed very similar trend in the effect on viability with high antigen doses (**Figure 6A**) as well as induction of all the previously described markers of activation, namely CD69 (**Figure 6B**) and downstream cytokine secretion (data not shown). When measuring PD-1, however, we were able to demonstrate the target-antigen HER2-dependent inhibition of PD-1 gene upregulation by our dCAS9-KRAB platform in RB-340 cells compared to the significant PD-1 induction in the conventional CAR-T cells (**Figure 6C**). In summary, using the aaTPs, we were able to fully recapitulate the expected platform activation in RB-340 and evaluate its potency in comparison to another benchmark cell product, which serves as a definitive proof for the suggested mechanism of action of a novel cell therapy product.

## Discussion

In physiology, T cells are effectively activated by professional antigen presenting cells (APCs) such as dendritic cells (DCs). An effective activation of T cells is mediated by the primary signaling conferred by the T cell receptor recognizing its cognate peptide presented by major histocompatibility complex (MHC) molecules and the secondary co-stimulation signaling conferred by interactions between various co-stimulatory receptors and their ligands. In order to induce such activation and resulting expansion of T cells for adoptive T-cell therapy without relying on autologous APCs, various designs of artificial antigen presenting cells (AAPCs) have been developed and used (20–24). The commercially available superparamagnetic microbeads that are coated with anti-CD3 and anti-CD28 antibodies (Dynabeads™ T-Activator) are one of the most widely used examples of AAPC in the current field of adoptive T cell therapy.

Unlike the non-engineered T cells, the CAR-T cells possess unique capability to be fully activated by mere recognition and binding of ectodomain of CAR to its epitope, which is enabled by the design of CAR. The designs of CAR have evolved from the first generation that contains only the intracellular signaling domain of CD3ζ for the primary (TCR) signaling to the second and the third generation designs to contain one or two additional co-stimulatory signaling domains derived from either the CD28 family (e.g. CD28 and ICOS) or the tumor necrosis factor receptor (TNFR) family (e.g. 4-1BB, OX40, or CD27), respectively (17). For CAR-T cells equipped with the second generation or later CARs, a simplest design of artificial target particles that only present the antigenic epitope on the surface of the selected biomaterial platform is possible. Despite the simplicity, to the best of our knowledge, no systematic effort to develop and use such artificial target particles for the development and manufacturing of CAR-T products has been previously reported.

Here we developed acellular artificial target particles (aaTPs) based on the commercially available biomaterial platform, Dynabeads™, to present HER2 tumor antigens to anti-HER2 CAR-T cells. We demonstrated that the HER2 surface densities on aaTPs can be precisely and systematically varied for a wide range. In this work, we fully standardized the preparation and characterization process of aaTPs. The HER2 loading efficiency were affected by the absolute concentrations of HER2 and microbeads in the incubation suspension, volume of the incubation suspension, incubation buffer, incubation time, and incubation temperature. But all of these parameters can be optimized, and once they are determined, the same conditions can be maintained constant as a standardized procedure (see Materials and Methods for the detailed conditions employed in this report). We also employed a highly standardized flow cytometry assay to determine the physiologically-relevant HER2 densities (ABC values) on the aaTPs.

Based on the tremendous success in the treatment of liquid tumors using CAR-T cells over the past decade, new CAR-T therapies with enhanced safety and efficacy are being developed and extending its territory to solid tumors (25). However, most of solid tumors do not express truly tumor-specific target antigens but rather only overexpress tumor-associated antigens (TAAs), and the expression levels of TAAs in solid tumors are very heterogenous.

Therefore, it is preeminent to precisely evaluate the sensitivity of CAR-T cells against target cells expressing varying densities of antigens, which enables further development of strategies to maximize the therapeutic window of CAR-T therapies for effective and safe treatment of solid tumors (4).

So far, systematic investigations for the sensitivity of CAR-T cells on the antigen densities have been performed using transformed target cell lines. The target cell lines were either retrovirally transduced to express various levels of antigens (3, 5) or selected and screened among various cell lines that intrinsically express various levels of the chosen antigen (26). In any case, such engineering of cell lines and clonal selection process are very time-consuming and expensive. More importantly, using various cell lines of different genetic background for further engineering as customized target cells for *in-vitro* evaluation of CAR-T cells makes it very difficult to reproduce the assays.

Here we propose the aaTP design as an alternative for standardized *in-vitro* assays of CAR-T cells. We investigated the activation of a second-generation anti-HER2 CAR-T cell by co-incubation with aaTPs presenting HER2 at varying densities at varying beads (aaTPs)-to-CAR-T-cells ratio. Indeed, antigen-specific activation of CAR-T cells was successfully induced by aaTPs, and the quantity of activation measured by expression of activation makers and secretion of cytokines showed clear antigen-dose dependency. Moreover, we also demonstrated that the aaTP design can be applied as a clean *in-vitro* assay tool for mechanism of action study of a next-generation CAR-T product.

Altogether, the aaTP design described here is very simple, inexpensive, modular, and easy to manufacture and maintain. Most importantly, these characteristics enable a development of standardized *in-vitro* assays for CAR-T products using conventional flow cytometry and cytokine measurement for the quantitative evaluation of critical attributes such as CAR-T sensitivity to varying antigen densities without having to engineer/manipulate biological model cell lines. Another significant benefit of the aaTPs over the conventional biological target cell line is that the same *in-vitro* assay samples from co-incubation of aaTPs and CAR-T cells can be directly used for subsequent genetic and epigenetic high-throughput sequencing analyses without concerns of contamination from the biological target cells. As the aaTP design using a commercially available magnetic bead platform is very versatile, it can be easily applied to other target antigens or extended to include other molecules relevant to the physiological target cells in the tumor microenvironments such as PD-L1. Overall, the aaTP design possess a great potential to be widely employed as a standardized tool for development and evaluation of conventional and next-generation of CAR-T products especially in the context of approval from regulatory agencies and clinical translation.

## Data availability statement

The original contributions presented in the study are included in the article/**Supplementary Materials**. Further inquiries can be directed to the corresponding authors.

## Author contributions

RH-S, data curation, formal analysis, investigation, methodology, validation, and writing draft. VSSAA, data curation, formal analysis, and investigation. LC, data curation and investigation. FM, investigation, resources, supervision, and revising draft. K-HR, conceptualization, formal analysis, investigation, methodology, validation, project administration, resources, supervision, writing, and revising draft. All authors contributed to the article and approved the submitted version.

## Conflict of interest

Authors RH-S, LC, and FM were employed by the company Refuge Biotechnologies.

The authors declare that this study received funding from Refuge Biotechnologies. The funder had the following involvement in the study: the study design, collection, analysis, interpretation of data, and the writing of this article.

## Publisher’s note

All claims expressed in this article are solely those of the authors and do not necessarily represent those of their affiliated organizations, or those of the publisher, the editors and the reviewers. Any product that may be evaluated in this article, or claim that may be made by its manufacturer, is not guaranteed or endorsed by the publisher.
