# Supplementary material for: Standardized in-vitro evaluation of CAR-T cells using acellular artificial target particles
Source: Front Immunol. 2022 Oct 20;13:994532. doi: 10.3389/fimmu.2022.994532 (PMC9632174; doi:10.3389/fimmu.2022.994532)
Supplement: Supplementary file 1 [file DataSheet_1.pdf]

Supplementary Material

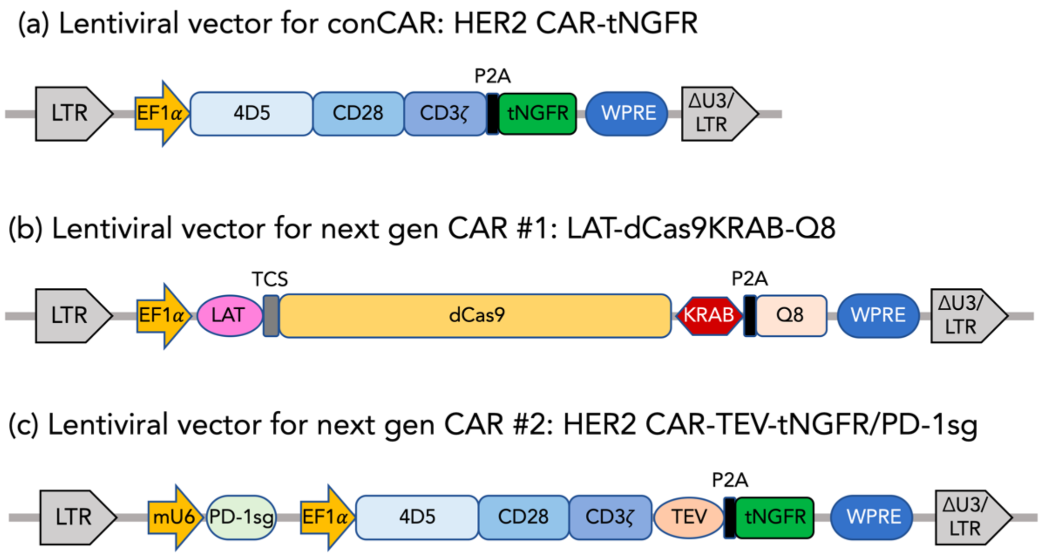

Supplementary Figure 1. Lentiviral components of a conventional anti-HER2 CAR T cell (a) and a next generation anti-HER2 CAR-T cell RB-340 (b and c). The CAR construct in (a) and (c) includes anti-HER2 (4D5 clone) scFv is combined to the CD28 and CD3ζ co-stimulatory domains. The tNGFR marker is connected via P2A. In (c), TEV is connected to CAR in tandem, and PD-1sg targeting the TSS of the endogenous PD-1 gene is included under mU6 promotor. In (b), LAT is connected to dCas9-KRAB via a TEV-cleavable sequence (TCS). The extracellular tag Q8 is connected via P2A.

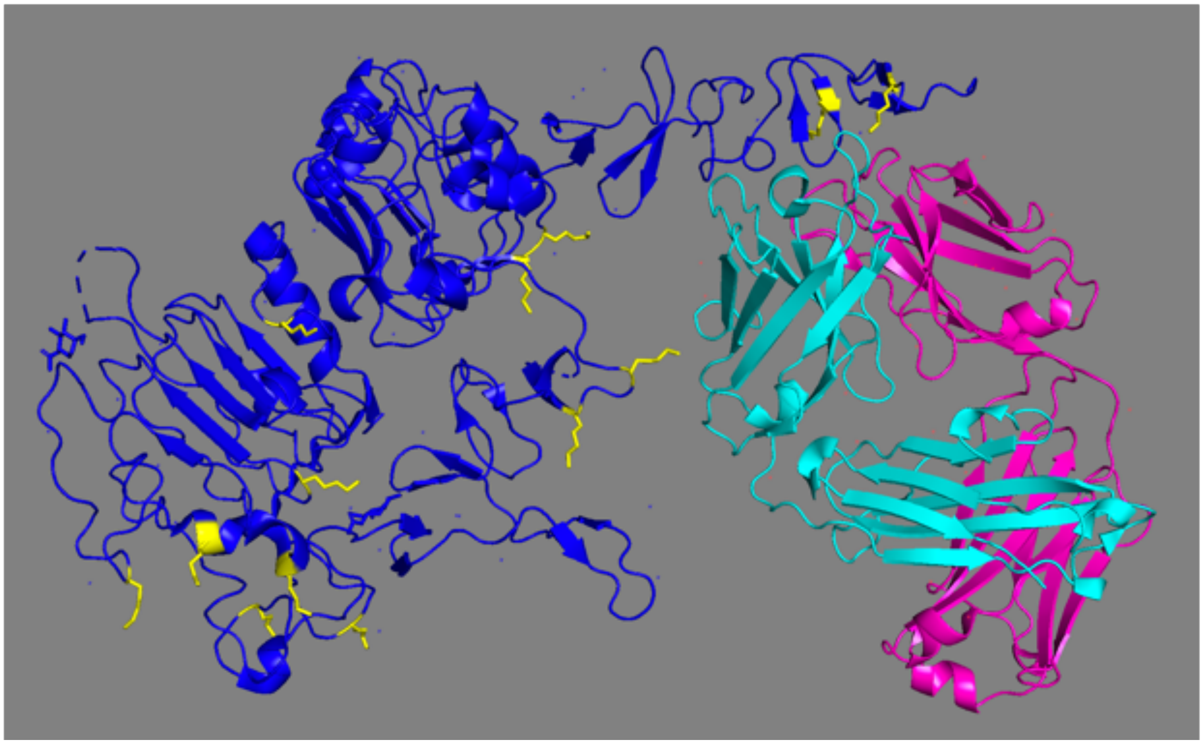

Supplementary Figure 2. Crystal structure of human HER2 (in blue) complexed with 4D5 Fab (in cyan and magenta, PDB ID: 1N8Z) rendered by using PyMOL. 13 lysine residues in the extracellular domain of human HER2 protein is depicted in yellow color.

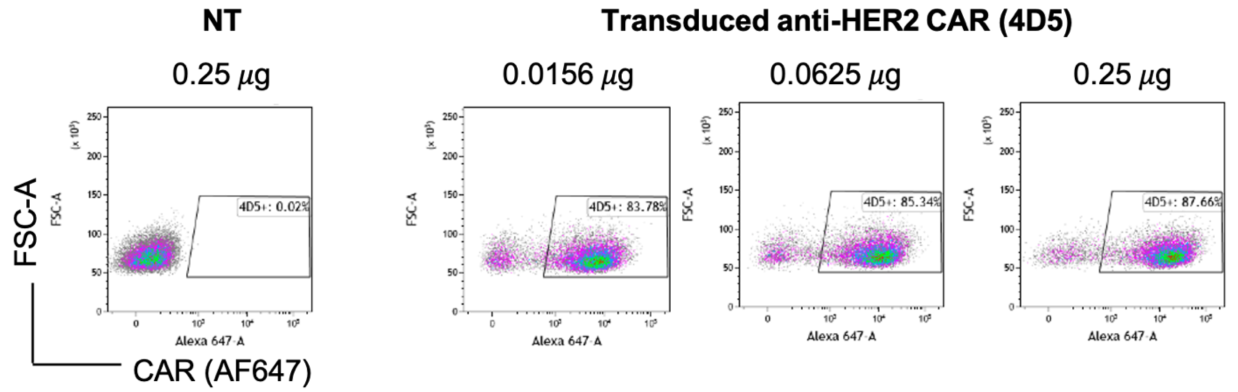

Supplementary Figure 3. Characterization of anti-HER2 CAR (4D5) expression by flow cytometry. The CAR-T cells were stained by recombinant human HER2 conjugated with Alexa Fluor 647 (AF647). The conjugation reaction between the HER2 and AF647 was performed by randomly targeting primary amine side groups of lysine residues on HER2.

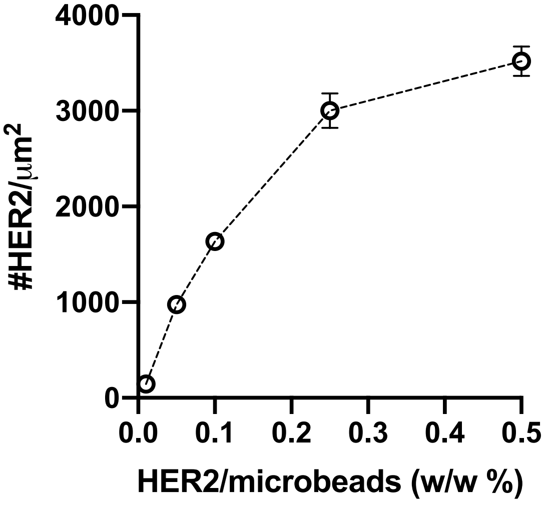

Supplementary Figure 4. Correlation between the loading condition (HER2/microbeads, w/w%) and resulting surface density (number of HER2 molecules per μm^2). The surface density represents antigen binding capacity (ABC) value measured by QIFIKIT®. Each data point represents mean ± standard deviation of 3 independent measurements.

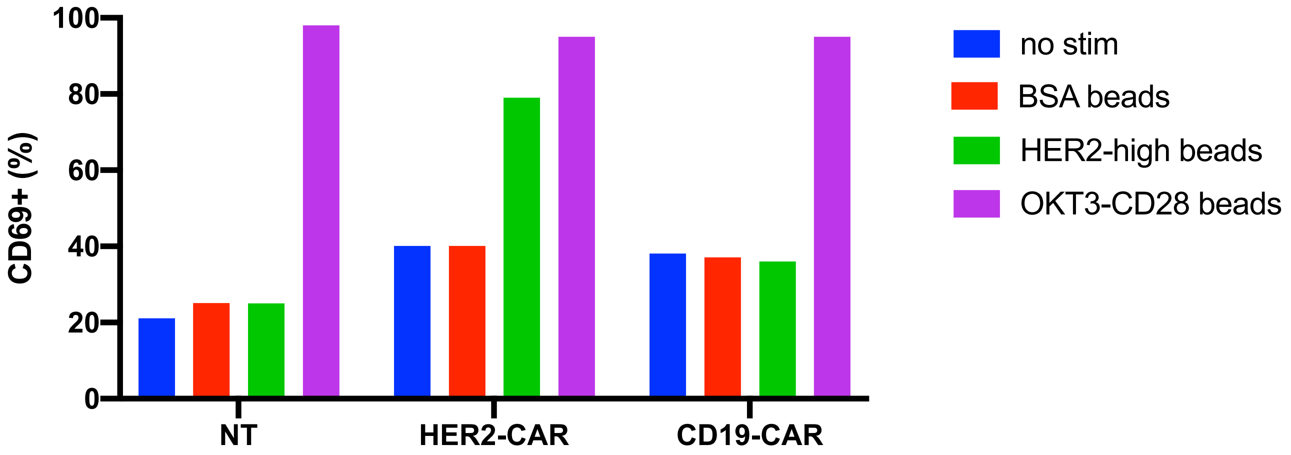

Supplementary Figure 5. Expression of activation marker CD69 on non-transduced T cells (NT), anti-HER2 CAR-T cells (HER2-CAR), and anti-CD19 CAR-T cells (CD19-CAR). The cells were incubated without any stimulation (no stim) or microbeads coated with BSA only (BSA beads) or HER2-high beads, or microbeads coated with anti-CD3 and anti-CD28 antibodies (OKT3-CD28 beads) at bead-to-CAR-T-cell ratio of 1:1 for 24 hours before examination by flow cytometry.
